# Phenotypic parameters affecting reproduction and production performances of dairy cattle in peri-urban of Bamako, Mali

**DOI:** 10.14202/vetworld.2019.817-822

**Published:** 2019-06-14

**Authors:** Abdoulaye Toure, Nicolas Antoine-Moussiaux, Fikremariam Geda, Ali Kouriba, Diakaridia Traoré, Bakary Traore, Pascal Leroy, Nassim Moula

**Affiliations:** 1 Department of Veterinary Management of Animal Resources, Fundamental and Applied Research for Animal and Health (FARAH), Faculty of Veterinary Medicine, University of Liege, Boulevard de Colonster, 20, Building B43, 4000 Liege, Belgium; 2 National Committee for Agricultural Research (CNRA), Bamako, Mali; 3 Laboratory of Research in Microbiology and Microbial Biotechnology (LaboREM-Biotch), Department of Biology, Faculty of Sciences and Techniques, University of Sciences, Techniques and Technologies of Bamako, Bamako, Mali

**Keywords:** cattle, Mali, milk production, peri-urban, reproduction

## Abstract

**Aim:**

The present study was conducted to evaluate the reproduction and production performances of crossbred and local dairy cattle in peri-urban of Bamako, the capital of Mali.

**Materials and Methods:**

A total of 17 randomly selected households with 450 dairy cattle from four peri-urban of Bamako were individually interviewed, given register format and divided into four focus group discussions. The participants were dairy farmers and they were asked to know the phenotype that seems to them the more interesting for the reproduction and production performances of their dairy cattle.

**Results:**

The calving interval (CI) obtained exceeded 433 days in all phenotypic types with average milk yields of 5.13±1.84 kg/day, 4.76±2.41, and 3.05±1.32 kg/day, respectively, for the Holstein crossbred, Montbeliard crossbred, and the local breeds (Zebu Peul and/or Zebu Maure). Crossbred was more productive than local breeds with significant (p<0.05) differences for lactation length, CI and total production and not significant on parity. The results also showed the advantages of the crossbred cows in terms of CI (460±80 days) compared to local zebus breeds (433±115 days) to keep the time of milking as long as possible. However, the Zebu Azawak breed whose cradle is located in Northern Mali, managed under extensive peri-urban rearing conditions, has less favorable production parameters than those of other local cattle breeds in milk production (636±43.3 kg vs. 681±41.1 kg).

**Conclusion:**

The present study revealed that crossbred dairy cattle performed better in terms of CI, lactation length, and production compared to the local breeds. The study also showed that the local Azawak breed numerically performed less compared to the other local breeds evaluated in this study, namely, Peul and Maure.

## Introduction

In Mali, the cattle breeds are currently mixed with exotic breeds either through artificial insemination or by crossing with bulls such as Montbeliard, Steppe Red, and Holstein. Crossbreeding optimizes the additive genetic and non-additive (heterotic) breed effects of *Bos taurus* and *Bos indicus* with consistently improving the lactation milk yield and calving interval (CI) with increasing European gene fraction, for at least up to 50% [1]. Despite the dairy potential that can be mobilized throughout the livestock sector and the main policies and strategies promoting the dairy sector, Mali remains one of the largest importers of dairy products to meet the needs of consumers. The import represents CFA 15 billion (the US $ 30 million) in milk and milk products to cover the needs of its population.

To cope with the deficit in milk production in Mali, exotic cattle breeds are increasingly imported and contributed to the genetic improvement of the local breeds. However, due to lack of sufficient knowledge of available genotypes, several cattle productivity improvement in the peri-urban area of Bamako has failed. The consequence of the situation could be the existence of a multitude of crossbred whose genotypes are not always known only on the basis of their phenotypes. Some studies were undertaken on phenotypic characterization and evaluation of dairy cattle for milk production performances [2,3].

However, studies in several developing countries emphasize that European breeds can only be profitable in a more intensive system that is favorable in terms of housing, food, and sanitary conditions [3-7]. Today, it becomes essential to evaluate the phenotypic parameters of dairy cattle in Mali, particularly the herds in the peri-urban area of Bamako to generate better knowledge and more rational use of crossing for sustainable milk production.

Therefore, the present study was conducted to evaluate the reproduction and production performances of crossbred and local dairy cattle in peri-urban of Bamako, the capital of Mali.

## Materials and Methods

### Ethical approval and Informed consent

Ethical approval is not necessary for such type of study. Informed consent was obtained from all participants involved in this study.

### Study area

**Figure-1:**
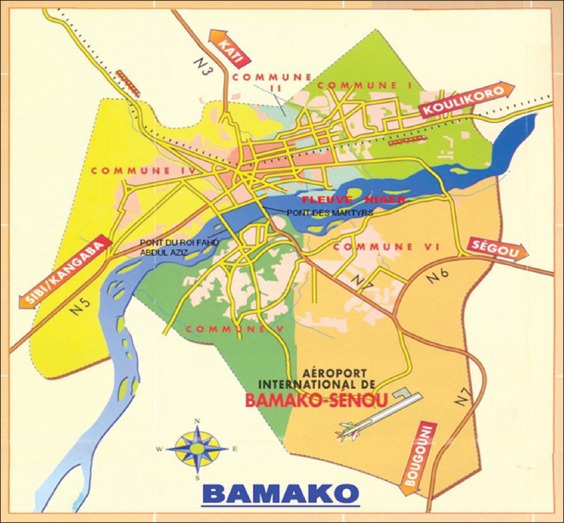
Map showing four peri-urban sampling regions of Bamako indicated by arrows and connecting the major regions of Mali (Source: http://www.mappery.com/maps/Bamako-Map.jpg).

The study was conducted in four peri-urban areas of Bamako, the capital of Mali (a sub-Saharan country in West Africa) covering the main road networks to other major regions (Ségou, Sikasso, Bougouni, Koulikoro, and Sibi/Kangaba road to Guinea) (Figure-1). Bamako is located on the Niger River (12°39’ N, 8°0’ W) and belongs to the Sahelo-Sudanian climatic zone, with a dry period from November to April and a wet period from June to October. The area has rain patterns of higher inter-annual variability. This study was carried out within a 100 km radius around Bamako, taking into account the established empirical estimates of Scholt *et al*. [5]. In this study, sampling was performed in ten selected peri-urban districts of Bamako (Bananko, Farakob, Fougad, Kabala, Kabalan, Moribab, Tienfal, Titib, Tiomadi, and Toubana) based on their potentiality in having a large number of cattle.

### Sampling procedure

A participatory approach survey involving focus group discussions (FGD) and visual tools was conducted on a total of 17 dairy farmers, with herd monitoring records in the period January 2012-December 2015. The farmers were identified through administrative services, dairy cooperatives, and accessibility. The selection was also based on the stability levels of their herds (installed for at least 10 years and have a fixed breeding nucleus), and the sites are easily accessible from the main road networks to the major administrative regions of Mali. The dairy farmers were grouped into four FGD and they had a total of 450 dairy cattle. The focus groups were made up of 94% men or one woman out of seventeen. Besides the register, the farmers were asked to know the phenotype that seems to them the more interesting for the reproduction and production performances of their dairy cattle. The crossbreds were grouped without taking into account the crossing level and the origin of the sire (artificial insemination or natural reproduction). For data collection, livestock technician and shepherd who could read and write data in the register and had easy road access were identified and trained as interviewers and supervised during data collection. The data were collected after visiting the farms by the interviewers and a focus group interview with local breeders or at the time of the deposit of milk to the cooperative. At least one sample was available in each direction indicated in Figure-1.

### Statistical analysis

To access different trends in the studied parameters, the collected data were subjected to a basic statistical analysis (frequency, calculation, mean, standard deviation, minimum, and maximum). The mixed procedure was used to compare the reproduction and production performances between breeds. Least squares means and standard deviation were calculated for all parameters. This analysis was performed using SAS software (SAS Institute, Inc., Cary, NC., USA) [8].

## Results

### Reproduction and production parameters

The phenotypic parameters of reproduction and production performances of cows are summarized in Table-1. The median dairy production for the 450 cattle studied was 895.5 kg, the minimum and maximum being 122 and 3267 kg, respectively, for 50 and 420 days of lactation.

**Table-1:** Reproduction and production performance parameters (LS means±SD) of dairy cattle (Holstein crossbred, n=86; Montbeliard crossbred, n=239; Azawak zebu, n=25; other local breeds (Peul and Maure), n=100) in peri-urban of Bamako.

Parameters	Holstein crossbred	Montbeliard crossbred	Azawak zebu	Peul and Maure	p-value
CI (days)	460±80^a^	443±97^b^	450±93^c^	433±115^d^	0.041
LL (days)	235±88^a^	229±85^b^	192±87^c^	221±79^c^	0.021
DMY (kg)	5.13±1.84^a^	4.76±2.41^a^	3.17±1.31^b^	3.05±1.32^b^	0.037
TPML (kg)	1210±60.4^a^	1065±54.9^b^	636±43.3^c^	681±41.1^c^	0.001
Parity	2.99±1.52	3.15±1.85	2.92±2.10	3.35±1.96	0.78

n=number, CI=calving interval, LL=lactation length, DMY=daily milk yield, TMPL=total milk production per lactation, LS=least squares, SD=standard deviation

### CI

**Figure-2:**
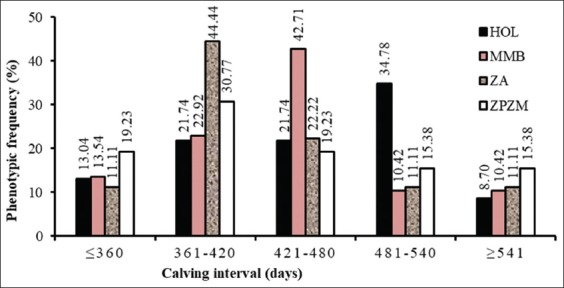
Distribution of calving interval frequencies of the declared phenotype (MHOL=Holstein crossbred, MMB=Montbéliard crossbred, ZA=Zébu Azawak, ZPZM=Peul and Maure Zebu).

The CI of Holstein varied from 297 to 618 days, with an average of 460±80 days (Table-1, Figure-2). These intervals were significantly (p<0.05) shorter than those of the local breed, which ranges from 331 to 658 days with an average of 433±115 days. Moreover, this interval remains non-significant for Montbeliard crossbred (298 and 707 days). This value was very high in previous researches in Holstein breed cows [3,6].

### Lactation length

The lactation length varies from 210 days to 420 days for cow survival without any other form of unforeseen such as the sale or death of the calf and the sale of the mother (Table-1, Figure-2). However, this duration is shorter in Zebu Azawak than in the other local breeds (Peul and Maure).

### Milk production

**Figure-3:**
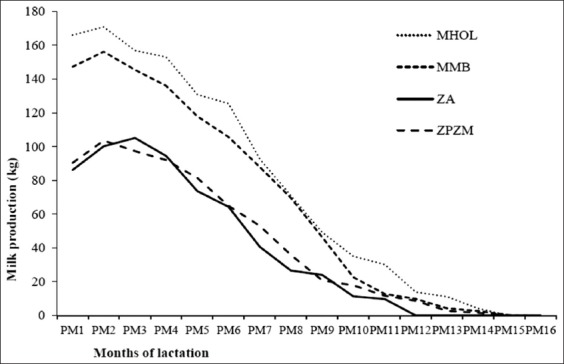
Lactation curve according to the declared genetic type (MHOL=Holstein crossbred, MMB=Montbéliard crossbred, ZA=Zébu Azawak, ZPZM=Peul and Maure Zebu).

The average daily milk production of milk per day and lactation of Holstein crossbred was significantly (p<0.05) higher than local Zebu and Maure breeds (Table-1, Figure-3). However, these two crossbred cows (Holstein and Montbeliard) showed a non-significant difference between the average annual milk yield and lactation length (Table-1). The milk yields varied from 3 to 12 kg/days and 586 to 3492 kg/lactation for Holstein crossbred, from 2.5 to 13.8 kg/days for Montbeliard crossbred. These values were lower than pure milk production (5606 kg) of Holstein cows previously [7].

## Discussion

### Dairy production and herd management

The choice of an adequate dairy system is relative to the individual need of the farmer and the optimal interaction between the physiological regulation of the animal and the technical skill of the farmer. The equipment and the quality of the work provided by the farmer used in the process of harvesting milk have been notified by other authors [9-11]. The milk production of the local Zebu Azawak breed was significantly different from the other local breeds (Zebu Peul and Zebu Maure) under the same breeding conditions. This could possibly due to not only a non-adaptation to better southern condition but also the physiology of the animal adapted to Sahelian conditions [12,13]. The superiority of Holstein and Montbeliard crossbreds, respectively, of 2210±548 kg/lactation versus 681±411 kg compared to the local breeds has been confirmed over several years and under the same farming conditions (Figure-3) [14]. The average dairy production per day is lower than that of Giroland cattle (6.0±0.2-8.7±0.2 kg/days and 1367±48-2230± 48 kg/lactation) in the Benin station [15]. It was the same as that obtained with F1 Ankolé X Sahiwal (4 kg/day) in Burundi [16]. The superiority of crossbreds was preferred by breeders who think that individuals from the cross of F1 with a crossbred adapt without difficulty to the breeding conditions of the area. Although the phenotype is the expression of the genotype influenced by the environment [3,5,6,17], the production objectives of the peri-urban livestock farmers in Bamako fall within a logic trend of dairy improvement with the various exotic breeds.

On the station, research conducted at the Sotuba Zootechnical Center in Mali has observed average animal production of females from the Zebu Peul-Montbeliard cross in the order of 1012 kg of milk as an average lactation of 243 days [18]. Studies in tropical countries have shown that 50% of blood Friesian produced more milk than 75% Friesian [19-21].

Adaptation to the local conditions is necessary for a balance between the breed potential toward optimum productivity. It is at this level that the individual objectives of the breeders are played out (the existence of an optimum). This option needs further investigation both by selection and by crossing.

### Reproduction and CI

Breeders have some expertise in cattle breeding, but management improvement is possible. They know that unexperienced bulls need accompanying during coitus. The breeders also know the bull to cow ratio, but there was only one breeding bull for a herd of 20-30 cows. This ratio thus reported involves the effectiveness of the bull in providing the irregular fertilizing projection. Some authors [2,22] have reported factors affecting the sex ratio such as year, season, bull, as well as blood hormone levels reported by Lari [23]. The average CI was 460±80 days for the Holstein crossbreds against 443± 97 days for the Montbeliard crossbreds. At 42.41% of CIs occurred between 421 and 480 days, Montbeliard crossbred against 34.78% between 481 and 540 for the Holstein half-breeds. Indeed, the CI and first insemination have not been calculated because most cows were not systematically inseminated at 90 days after calving.

The longest CI for the crossbreds could be due to a guaranteed purchase of milk by cooperatives but also the managerial capacity of a farmer, for instance in providing appropriate diet [24] and according to the season of birth [25]. However, this long CI reduces the annual production cycle, and the amount of milk a cow could likely give during a period [26].

The reproductions characterized globally by an absence of control of the coitus, especially in community grazing land. This phenomenon, in the context of the practice of crossbreeding by some breeders to negative externalities (purity of their herd) for reasons of hardness and undermines the sustainability of the systems through an uncontrolled system increase on the parts of genetics [27]. Thus, the interval between the ages showed strong variation and was neither shorter than that observed on the cows in Kenya which was an average 646 days [28] and 477±119 days nor than the CI in Tchad [29]. However, these values are improved, compared to the parental breed (N’Dama 300-822 days) at the Yamoussoukro breeding station [17].

Exchange practices or purchases of external breeders were also observed in the present study. The low turnover rate of breeding bulls may lead to some inbreeding in the herd. The high number of genetic profile reveals the absence or non-mastery of crossing patterns appropriate to achieve a high performing synthetic breed adapted to local conditions. This would require the involvement of public authorities in the dynamic process of innovation: Breeders are trying.

The breeders are under various influences and must be framed in obtaining and taking into account their specific objectives. “A soft” approach to biodiversity conservation can be adapted with the goal of not keeping local breeds as they are, but ensure that the animals in the world are not all the same and that some particularly interesting characters are not lost.

### Optimal milk production strategy

Three main trends emerge in this strategy, namely the poor conservatives whose opinion is probably related to their living conditions; evolutionists/enhancers and farmers with no tendencies between the first two strategies. The performance of the crossbred is superior to that of their parents due to the heterosis effect [30-32]. Breeders, as part of their breeding goals, know this strategy. The dairy trend, as expressed in the previous study [32], shows an interest in cattle breeding and may explain the choice of certain breeders for the practice of crosses. If these crosses allow an improvement in the quantity of milk, they nevertheless contribute to reducing the adaptation to environmental stresses of the animals [7]. The breeding practices thus observed are presented as a compromise according to the objectives to make the best use of the animal resources available to the breeder.

Several options could be available for increasing milk productivity and developing smallholder and large-scale herds. However, farmers faced major technological and environmental challenges in the current production as described elsewhere [33-35]. These challenges need government intervention.

## Conclusion

The present study revealed that crossbred dairy cattle performed better in terms of CI, lactation length, and production compared to the local breeds. The study also showed that the local Azawak breed numerically performed less compared to the other local breeds evaluated in this study, namely, Peul and Maure.

## Authors’ Contributions

NA, PL, and NM conceived and designed the study. AT and BT surveyed under the guidance of AK and DT. AT, FG, NA, and NM drafted and revised the manuscript. All authors read and approved the final manuscript.

## References

[1] Kahi A.K, Nitter G, Thorpe W, Gall C.F (2000). Crossbreeding for dairy production in the lowland tropics of Kenya:II. Prediction of performance of alternative crossbreeding strategies. Livest. Prod. Sci.

[2] N'Goran K.E, Zakpa L.G, Noel D.D, Lallié H.D.M, Sokouri D.P, Doumbia L (2016). Production and reproduction parameters analysis of N'Dama cattle breed in the dairy station of Yamoussoukro (SLY), in the savannah zone, in Côte d'Ivoire. Int. J. Res. Rev.

[3] Çilek S (2009a). Reproductive traits of Holstein cows raised at Polatli state farm in Turkey. J. Anim. Vet. Adv.

[4] Kumar N, Abadi Y, Gebrekidan B, Woldearegay Y.H (2014). Productive and reproductive performance of local cows under farmer's management in and around Mekelle, Ethiopia. J. Agric. Vet. Sci.

[5] Scholt M.M, Theunissen A (2010). The use of indigenous cattle in terminal cross-breeding to improve beef cattle production in Sub-Saharan Africa. Anim. Gen. Res.

[6] Bakir G, Cilek S (2009). A research on reproductive traits of Holstein cattle reared at Tahirova state farm in Balikesir province in Turkey. J. Anim. Vet. Adv.

[7] Çilek S (2009b). Milk yield traits of Holstein cows raised at Polatli state farm in Turkey. J. Anim. Vet. Adv.

[8] SAS (2001). Statistical System Institute, User's Guide:Statistics.

[9] Delgado C, Rosegrant M, Steinfeld H, Ehui S, Courbois C (1999). Livestock to 2020:The Next Food Revolution. Food, Agriculture and the Environment. Discussion Paper, No. 28. IFPRI/FAO/ILRI.

[10] Genzebu D, Tamir B, Berhane G (2016). Study of productive and reproductive performance of cross breed dairy cattle under smallholders'management system in Bishoftu and Akaki towns. Int. J. Agric. Sci.

[11] Hatungumukama G, Sidikou D.I, Leroy P.L, Detilleux J (2009). Effects of non-genetic and crossbreeding factors on dairy milk yield of Jersey x Sahiwal x Ankolécows in Burundi. J. Anim. Vet. Adv.

[12] Orgeur P, Signoret J.P (1990). L'activitésexuelle du taureau:Revue bibliographique. INRA Prod. Anim.

[13] Tellah M, Zeuh V, Mopaté L.Y, Mbaïndingatoloum F.M, Boly H (2017). Paramètres de reproduction des vaches Kouri au Lac Tchad. J. Appl. Biosci.

[14] Coulibaly M.D, Traoré A, Cissé A.B, Traoré D, Ouologuem B (2004). Amélioration de la Productivitédes Races Bovines Autochtones par le Croisement, Performances Laitières des Croisés Rouge des Steppes. Etudes et Recherches Saheliennes Institut Sahel, No. 8-9.

[15] Dalcin V.C, Fischer V, Daltro D.S, Alfonzo E.P.M, Stumpf M.T, Kolling G.J, Silva M.V.G, McManus C (2016). Physiological parameters for thermal stress in dairy cattle. R. Bras. Zootec.

[16] Gezu T, Moges D (2018). Impact of climate change on smallholder dairy production and coping mechanism in Sub-Saharan Africa - review. Agric. Res. Technol.

[17] Lari M.A (2006). Sex ratio at birth in dairy herds in Fars province, Southern Iran. Trop. Anim. Health Prod.

[18] Bassirou B (2002). Lait Sain Pour le Sahel. Atelier de Restitution des Résultats Hygiène et Qualitédu lait et des Produits Laitiers au Mali:Implications en Production Laitière et en SantéPublique.

[19] Ba Diao M, Dieng A, Seck M.M, Ngomibé R.C (2006). Pratiques alimentaires et productivitédes femelles laitières en zone périurbaine de Dakar. Rev. Elev. Méd. Vét. Pays Trop.

[20] Ahmed F.A, Babiker B.A, Mohamed T.M, Ali T.E (1992). The effect of genetic upgrading of Kenana (Sudan Zebu cattle) with European Friesian on calf performance, milk yield and milk composition. Rev. Élev. Méd. Vét. Pays Trop.

[21] Leroy G, Baumung R, Boettcher P, Scherf B, Hoffmann I (2016). Review:Sustainability of crossbreeding in developing countries, definitely not like crossing a meadow. Animal.

[22] Hanuš O, Frelich J, Janů L, Macek A, Zajíčková I, Genčurová V, Jedelská R (2007). Impact of different milk yields of cows on milk quality in Bohemian spotted cattle. Acta Vet.

[23] Kassa S.K, Salifou C.F.A, Dayo G.K, Ahounou S, Dotché O.I, Issifou T.M, Houaga I, Koutinhouin G.B, Mensah G.A, Yapi-Gnaoré V, Youssao A.K.I (2016). Assessment of milk production and resilience of Girolando cattle, reared in semi-improved breeding system in Benin. J. Vet. Adv.

[24] Doko A.S, Gbégo T.I, Tobada P, Yari H.M, Lokossou R, Tchobo A, Alkoiret T.I (2012). Performances de reproduction et de production laitière des bovins Girolando àla ferme d'élevage de Kpinnou au sud-ouest du Bénin. Bull. Rech. Agron. Bénin.

[25] Haftu K (2015). Productive and reproductive performance of Holstein-Friesian cows under farmer's management in Hossana town, Ethiopia. Int. J. Dairy Sci.

[26] Abebe B, Zelalem Y, Ajebu N (2014). Dairy production system and constraints in Ezha districts of the Gurage zone, Southern Ethiopia. Glob. Vet.

[27] Tadesse B, Tassew M, Kefelegn K, Million T (2015). Estimation of crossbreeding parameters for milk production and reproduction traits in Holstein Friesian and Ethiopian Boran crosses. J. Reprod. Infertil.

[28] Madalena F.E, Teodoro R.L, Lemo A.M, Monteiro J.B.N, Barbosa R.T (1990). Evaluation of strategies for crossbreeding of dairy cattle in Brazil. J. Dairy Sci.

[29] Syrstad O, Ruane J (1998). Prospects and strategies for genetic improvement of the dairy potential of tropical cattle by selection. Trop. Anim. Health Prod.

[30] Kaygisiz A, Vanli Y (2008). Factors influencing sex ratio in Brown Swiss cattle. Indian J. Anim. Sci.

[31] Odima P.A, Mcdermott A (1994). Reproductive performance of dairy cows on smallholder dairy farms in Kiambu district, Kenya:Design, methodology and development considerations. Kenya Vet.

[32] Tadesse G, Mengistie A (2016). Challenges, opportunities and prospects of dairy farming in Ethiopia:A review. World J. Dairy Food Sci.

[33] Coulibaly M.D, Ouologem B, Togola D (2005). Recherche par Voie de Croisement de Génotypes Appropriés Pour la Production de Lait. Rapport Final de Recherche. 11^ème^Session du Comitéde Programme.

[34] Djoko T.D, Mbah D.A, Mbanya J.N, Kamga P, Awah N.R, Bopelet M (2003). Crossbreeding cattle for milk production in the tropics:Effects of genetic and environmental factors on the performance of improved genotypes on the Cameroon Western high plateau. Rev. Élev. Méd. Vét. Pays Trop.

[35] Sophie M.A.D (2009). Le Lait Local en Périphérie de Bamako:Une Filière en Sursis?Echo Géo.

